# Estimation of Ecotourism Carrying Capacity for Sustainable Development of Protected Areas in Iran

**DOI:** 10.3390/ijerph19031059

**Published:** 2022-01-18

**Authors:** Parvaneh Sobhani, Hassan Esmaeilzadeh, Seyed Mohammad Moein Sadeghi, Marina Viorela Marcu

**Affiliations:** 1 Environmental Sciences Research Institute, Shahid Beheshti University, Evin, Tehran 1983969411, Iran; p_sobhanipajoh@sbu.ac.ir; 2 Department of Forest Engineering, Forest Management Planning and Terrestrial Measurements, Faculty of Silviculture and Forest Engineering, Transilvania University of Brasov, Şirul Beethoven 1, 500123 Brasov, Romania; moeinsdgh@hotmail.com (S.M.M.S.); viorela.marcu@unitbv.ro (M.V.M.)

**Keywords:** Tehran, Delphi method, effective carrying capacity, physical carrying capacity, real carrying capacity, environmental sustainability, visitor management

## Abstract

Estimating the ecotourism carrying capacity (ETCC) in protected areas (PAs) is essential for minimizing the negative impacts of ecotourism and sustainable environmental management. PAs are one of the prominent ecotourism locations and many of these areas have been created to protect biodiversity and improve human wellbeing. This study has identified and prioritized negative impacts of ecotourism in Lar national park, the Jajrud protected area with the sustainable use of natural resources, and Tangeh Vashi national natural monument. For this purpose, physical carrying capacity (PCC), real carrying capacity (RCC), and effective carrying capacity (ECC) were estimated using the ETCC model. The results indicated that due to these areas’ ecological sensitivity, the most negative impacts of ecotourism are related to the environmental-physical dimensions. In contrast, the lowest impacts have been observed in the economic-institutional dimensions. Moreover, the results revealed that the highest PCC is related to Lar national park, and the lowest PCC is associated with Tangeh Vashi natural monument. There are more tourists in the Jajrud protected area with the sustainable use of natural resources than other areas in RCC and ECC due to low levels of restrictions and legal instructions. In contrast, in Lar national park and Tangeh Vashi natural monument, due to the short duration of ecotourism in these areas (from June to October), high level of restrictions, and ecological sensitivity, the number of tourists is less than the RCC and ECC. As these areas have a limited ability to attract visitors and ecotourism, the protection of these areas requires the implementation of sustainable management to control the negative impacts of ecotourism and estimate the number of visitors.

## 1. Introduction

Today’s expansion of ecotourism activities on a global scale has led to physical damage, an increase in pollution, landscape degradation, the destruction of flora and fauna, water shortages, and so on, especially in natural ecosystems and protected areas (PAs) [1,2]. PAs are identified as the most essential areas in the world, and many of these areas have been created as an ideal strategy to conserve biodiversity and improve human wellbeing [3,4]. Furthermore, PAs have various natural, historical, cultural, and ecotourism attractions and a high biological value for plants and animals [5,6]. Accordingly, ecotourism as a sustainable tool in PAs leads to a balance in biodiversity conservation, economic development, and improvement in the livelihoods of local people and communities [7]. Therefore, one of the goals for PAs is to protect natural resources and provide a space for environmental education and nature-based tourism activities [7,8]. Since increasing the number of tourists affects system flexibility and ecotourism satisfaction, PAs, as a strategy, can benefit from sustainable ecotourism to maintain the landscape, provide recreational services for visitors, and promote environmental education [2,9,10]. The excessive number of tourists in natural ecosystems and PAs has negative consequences for the quality of residents’ lives and the experiences of tourists [11,12]. Since these areas have a limited ability to attract visitors and ecotourism, the carrying capacity model is often considered in the framework for developing sustainable ecotourism [13,14]. A definition of tourist carrying capacity proposed by the World Tourism Organization (WTO) in 1981 was: “the maximum number of people that can visit a tourist destination simultaneously, without causing damage to the physical, economic, social, and cultural environment or an unacceptable decline in the quality of tourists’ satisfaction with their stay” [15]. As a result, the concept of carrying capacity, which includes all these aspects, is often used to develop sustainable tourism, thereby protecting the destination physically, socially, culturally, and ecologically [16]. Due to these areas’ biological potential and physical characteristics, some ecotourism destinations have suffered extensive negative impacts as a result of increased numbers of tourists [17,18]. Therefore, estimating the carrying capacity can be used to manage plans and develop sustainable ecotourism in these areas [13]. Accordingly, the ecotourism carrying capacity (ETCC) in these areas has been considered by managers, decision-makers, and stakeholders [1,19,20].

ETCC is an essential tool in ecotourism planning, which provides sustainable ecotourism standards and the protection of cultural heritage [21]. Hence, applying and estimating ETCC is a broad process of sustainable ecotourism planning that can help local communities, planners, and decision-makers provide an overall framework [19,20]. In addition, ETCC is associated with various components, including environmental-physical, social-demographic, and political-economic. Indeed, a protected and ecologically balanced environment is necessary to develop sustainable ecotourism based on carrying capacity analysis [22]. Therefore, the purpose of estimating ETCC is to provide a balance between environmental protection and sustainable management [19,23]. Since biodiversity is one of the main ecotourism attractions in these areas, it needs a tool to assess and manage the negative impacts of ecotourism on the environment [24,25].

There are several types of PAs, which vary in the level of protection, depending on the laws of each country or the regulations of the international organizations involved. According to the IUCN definitions [26], PAs can be classified into strict nature reserves, wilderness areas, national parks, natural monuments, habitat species management areas, protected landscapes/seascapes, and protected areas with the sustainable use of natural resources. The growing global demand for agricultural and forest resources has led to downgrading and downsizing PAs [27,28] to exploit their resources without being eroded by illegal harvesting [28,29]. In PAs, creating some activities is possible according to the rules and capacities. According to the above studies, the ETCC model has been used to protect the environment by balancing the number of tourists and attracting visitors. There is insufficient attention on identifying and classifying the related impacts of ecotourism in different dimensions and sustainable ecotourism management. Accordingly, this issue has been the focus of this study, and adverse effects of ecotourism in PAs have been identified and assessed in different dimensions, including environmental-physical, demographic-social, and economic-institutional with the participation of experts’ groups. Then, to reduce the negative impacts of ecotourism in these areas, physical carrying capacity (PCC), real carrying capacity (RCC), and effective carrying capacity (ECC) have been estimated.

Unfortunately, PAs that are part of biological reserves globally have been affected by environmental threats due to the expansion of ecotourism and excessive visitors. These impacts have led to increased unsustainability, habitat degradation, changes in landscape structure, and species extinction. Hence, the expansion of ecotourism is one of the significant threats in these areas. The PAs of Tehran province have natural, historical, and cultural attractions and vegetation and wildlife species with high biological value. Due to the mountainous climate, abundant springs, and water resources, these areas are considered one of the unique mountain ecosystems of southern Alborz. These areas have a high natural value due to scarce endemic species. Overuse of the PAs may disturb the fragile soils, vegetation, and wildlife and cause unacceptable crowding and visitor conflicts. Therefore, most PAs need visitor management to enhance their values, such as when ecotourism has become an integral component of the natural ecosystems.

## 2. Literature Review

There are few places in the world where ecotourism is as popular as the PA [6,30,31,32]. The WTO [33] emphasized the importance of planning, managing, and monitoring ecotourism operations for PAs to stay sustainable in the long term. It should be noted that ecotourism is one of the components of sustainable development, which can be achieved by integrating social, economic, and environmental resources [34,35,36]. In this regard, Honey [34] stated that ecotourism is distinguished as a pathway to sustainable development through which nature is supremely commodified without degrading the environment while supporting local peoples. Ma et al. [37] studied the sociodemographic characteristics of tourists on the travel motivation and satisfaction related to PAs in China. They found that education level was negatively correlated with the satisfaction of tourists. Aryal et al. [38] evaluated ecotourism-related policies in the PAs of Nepal and concluded that the tourist number in the PA was observed to increase in response to the growing number of tourists visiting this country.

Using the ETCC concept in PAs may generate a satisfactory experience for ecotourism with an acceptable or minimum impact on the resource of these areas [14]. An extensive body of literature indicates that studies have calculated the ETCC in natural environments and studied the influence of ecotourism on natural resources and PAs. For example, Tokarchuk et al. [39] estimated the tourism social carrying capacity in Berlin, Germany. They proposed a novel method of measuring the social carrying capacity threshold by measuring subjective well-being. They found that social carrying capacity can be estimated as the level of tourism intensity in the area where social well-being has decreased. Makhadmeh et al. [21] studied the carrying capacity at the archaeological site of Jerash (Jordan) using mathematical GIS modeling. The results revealed that the carrying capacity in the area is currently a severe challenge, and the excessive number of tourists and festival attendants put its archaeology in danger because the density of tourists is not uniform all over the site or throughout the year. Accordingly, more cooperation should be provided between decision-makers, stakeholders, tour operators, and the local community. Corbau et al. [15] studied tourism in Asinara Island (Italy) using carrying capacity and web evaluations. They found that tourism has many environmental impacts, particularly in marine PAs. Their analysis revealed that the tourism carrying capacity (TCC) in this area is lower, and the tourist experience was excellent. Chen and Teng [40] studied management priorities and carrying capacity at a high-use beach in Taiwan from tourists’ perspectives, as a way towards sustainable beach tourism. The results showed that beach cleanliness, safety, information provision, sediment and habitat management, and overcrowding were more important to tourists, indicating the priority of these measures in the area. Salerno et al. [41] proposed a new TCC concept from a more management-oriented perspective to operationalize sustainable ecotourism in PAs. Salemi et al. [23] evaluated the ETCC in Karkheh PA, in southern Iran, and concluded that the study area has a high ETCC, which can accept visitors. In a study by Wiyono et al. [42] in the Ujung Kulon national park of Indonesia, the PCC, RCC, and ECC were estimated as 20,000, 4838, and 6 visitors per day (v/d), respectively. Amiry Lagmoj et al. [43] estimated the ETCC using PCC, RCC, and ECC indices in the Khorma PA of Langeroud city, in northern Iran. The findings showed that the PCC, RCC, and ECC were estimated to be 3712, 2001, and 69 v/d, respectively. Jangra and Kaushik [44] predicted the ETCC by applying the PCC, RCC, and ECC formulas. They found that the PCC, RCC, and ECC of selected tourist spots in the Himalayan Mountains of India were 64,835, 9595, and 5928 v/d, respectively.

Ecotourism in the PAs of Iran has captured the attention of many sectors [4,23,45,46]. In the present study, in addition to assessing the state of the ETCC in the PAs through the carrying capacity model, the results obtained in different PAs have been compared. To the best of our knowledge, no studies have been performed in this regard. Hence, this study aimed to estimate the ETCC at three levels (i.e., PCC, RCC, and ECC) in Lar national park, the Jajrud protected area, with the sustainable use of natural resources (hereafter Jajrud PA), and Tangeh Vashi national natural monument. Consequently, the main questions of this research are: (1) What are the main negative impacts of ecotourism in PAs? (2) What are the PCC, RCC, and ECC in the studied areas? And (3) What is the status of the ETCC in the studied areas?

## 3. Materials and Methods

### 3.1. Study Area Description

**Figure 1:**
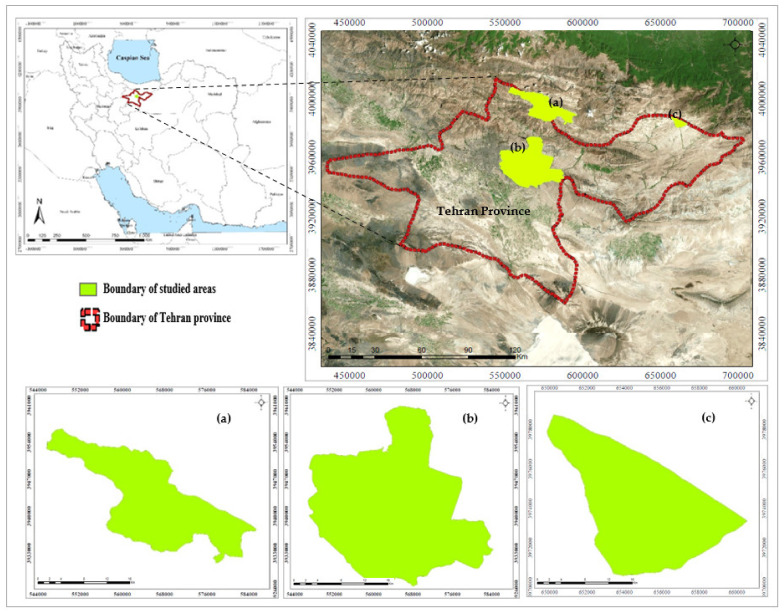
Location of the studied areas; (**a**) Lar national park, (**b**) Jajrud PA, and (**c**) Tangeh Vashi natural monument.

The province of Tehran covers 18,800 hectares and occupies the northern portion of Iran’s central plateau. This province has over 13.5 million inhabitants and is Iran’s most densely populated province. In general, this province has a semiarid climate with an average annual temperature of 17 °C and an annual precipitation of around of 250 mm [47,48]. The study areas are located in Tehran province, including Lar national park, Jajrud PA, and Tangeh Vashi national natural monument (Figure 1 and Table 1). Many ecotourism attractions, a high number of visitors, uncontrolled ecotourism development, unsustainability, and habitat destruction are visible in these areas. Hence, according to the mentioned factors, three areas have been studied, as follows:

(1) Lar national park, known as Lar plain, is one of the richest habitats of Tehran province. Due to the importance and necessity of its protection, it has now been declared a preservation area by the Department of Environment of Iran. With an area of 35,765 ha, this park is located between Tehran and Mazandaran provinces. Hence, one of the best views of the highest peaks of Iran can be seen from Lar Park, and due to the many natural attractions, such as Lar Lake, many springs and rivers are visited by a large number of visitors during the year. Moreover, 405 vascular plant species and 159 animal species have been known in this area. The predominant vegetation of the area is composed of pasture plants and grasslands. This area is also the habitat of unique aquatic species (i.e., brown trout (*Salmo trutta fario*)). As a symbol of biodiversity protection in Tehran province, the brown trout is known as one of the rarest marine species globally [49,50].

(2) Jajrud PA, with an area of about 74,811 ha, is another PA of Tehran province. The highest elevation in this area is Arakuh peak, with an elevation of 2600 m above sea level. Therefore, Jajrud PA is one of the most prominent habitats of wild sheep (*Ovis orientalis*) and wild goat (*Capra aegagrus*). This area has various appearances in terms of vegetation due to its high elevations. There are 517 vascular plant species that have been identified in the area. In addition, Khojir and Sorkheh Hesar national parks are located in this area with high flora and fauna. The region’s mountainous landscape and the Jajrood River, Kamard Waterfall, Shemshak and Dizin International Ski Resorts, and Hamloon Cave can attract tourists to the area. Hence, Jajrud PA is known as one of the oldest PAs in the world [51].

(3) Tangeh Vashi, with an area of 3650 ha, is one of the national natural monuments in this province. This area is a gorge and mountain pass in the Alborz slope, a popular ecotourism attraction in Tehran province. The vegetation known in Tangeh Vashi is mainly rangeland species, and galbanum (*Ferula galbaniflua*) is one of the dominant plant species in this area [52].

It should be noted that these three sites have a management plan. Figure 1 depicts the location of the studied area [53].

### 3.2. Methodology

#### 3.2.1. General Framework and Data

**Figure 2:**
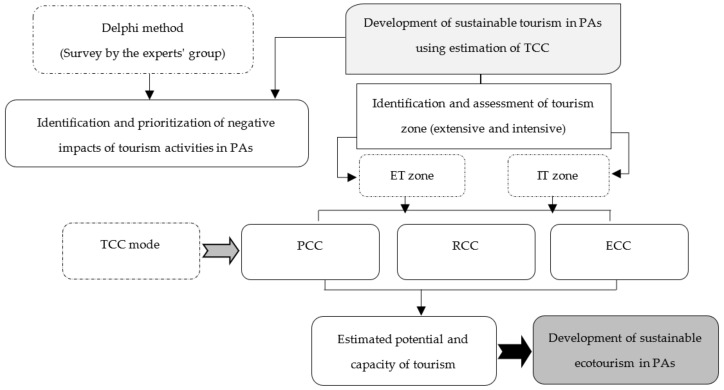
Diagram of the research methodology. PAs—Protected Areas; ET— Extensive Ecotourism; IT—Intensive Ecotourism; TCC—Tourism Carrying Capacity; PCC—Physical Carrying Capacity; RCC—Real Carrying Capacity; ECC—Effective Carrying Capacity.

According to Iranian experts‘ viewpoints, the present study analyzed the negative impacts of ecotourism in PAs using the Delphi method, then, identified and evaluated the ecotourism potential and capacity in Lar national park, Jajrud PA, and Tangeh Vashi national natural monument using the ETCC model. In these areas, annual visits and the geographical, biophysical, ecological, and managerial characteristics were investigated. Then, according to the national recreation classes for PAs, extensive ecotourism classes (ET) and intensive ecotourism classes (IT) were calculated [54,55]. Then, the PCC, RCC, and ECC were estimated using the Cifuentes method [56], which was suggested by the IUCN [57] and was widely accepted in past studies [58,59,60,61,62]. Based on the specific characteristics and peculiarities of a particular location, the Cifuentes method attempts to establish the maximum number of visits that the area can tolerate [44]. Figure 2 shows the flowchart of the methodological process.

#### 3.2.2. The Delphi Method

**Figure 3:**
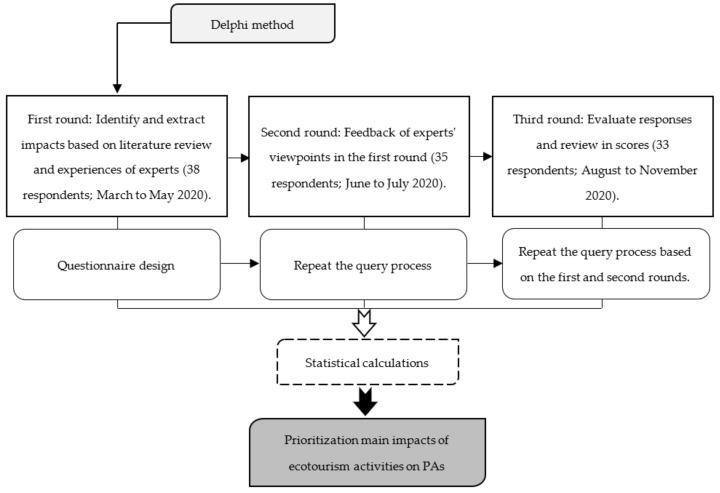
Main steps of the Delphi method.

The Delphi method has been used to capture group knowledge that helps decision-making during surveys, data collection, and final consensus [63,64,65]. In this method, researchers create a theoretical consensus based on expert groups, where data and information are unavailable and expert opinions differ [65,66,67]. The Delphi technique requires the careful selection of experts, as it reflects the quality of the decision made by the group [68,69]. Experts must be knowledgeable and aware of the issue or problem under consideration [70,71]. Letters of invitation for subject matter experts’ participation in this study were sent to academics and managers with related scientific specialties, such as environmental engineering, environmental pollution, tourism, geography, natural resources, biodiversity, forests, and geography in PAs in Iran. The managers must have at least five years of experience in ecotourism and related areas (such as management of PAs). The requirements for academic participants included five years of teaching experience in tourism at a university, tourism publications relevant to Iranian tourism, and an interest in tourism management research. The survey questionnaire was distributed through mail services, online, and face-to-face. Where necessary, we contacted them in person and by e-mail and telephone. Hence, this work opted for an initial (i.e., round one) panel size of 38 experts. The three-round survey took place between the end of March 2020 and the beginning of November 2020 (Figure 3).

This study used a survey questionnaire as the main instrument for implementing the Delphi method. The questionnaire about the impacts of ecotourism was prepared using a five-point Likert scale (1 = not at all influential, 2 = slightly influential, 3 = somewhat influential, 4 = very influential, and 5 = extremely influential). Therefore, to assess the impacts of tourism activities, 40 indicators were designed in a series of questionnaires to survey the opinions of 38 national experts, all from Iran. All the experts stated that they had visited the study areas at least once a year in the last five years. Moreover, they clarified that they have thoroughly studied the management plan, as well as the available literature for each site.

Moreover, the collected data was analyzed using statistical tests. Hence, the mean, standard deviation (SD), and variance (V) were calculated for each impact. To determine whether or not the answers differed widely among the respondents, we calculated the SD. Finally, according to the obtained results, the impacts of ecotourism were prioritized from high to low. The Delphi method was conducted through three rounds (Figure 3). The first round of the Delphi questionnaire was designed based on experiences and literature reviews. In the second round, according to the feedback of the experts in the first round, questioning was repeated. This round was held among 38 respondents, and 3 persons did not respond to the questions within the allowed time. Finally, in the third round, responses were asked to evaluate and score their initial responses. In this round, 2 persons did not respond to the questions, and 33 questionnaires were fulfilled to analyze and recognize the major impacts of ecotourism in the studied areas. The final list of experts had, on average, 21 years of experience working or researching with or for PAs (minimum six years; maximum 26 years). Using the Delphi method, the responses would remain anonymous to other panel members. After obtaining the final viewpoints of the members, statistical calculations and their prioritization were performed. Accordingly, the results of the Delphi method were obtained based on the feedback of experts. Then, by analyzing these viewpoints a consensus was reached on the possible impacts of ecotourism in PAs (Figure 3).

#### 3.2.3. Estimation of ETCC

ETCC has been used as a valuable tool to sustainable ecotourism development in PAs [62], which requires a deep knowledge of the studied areas and is based on the five fundamental following steps [61]:

- Analysis of ecotourism management policies in PAs.
- Calculation of expected targets in PAs.
- Analysis of the current ecotourism situation in studied areas.
- Identify the effective characteristics of PA management.
- Estimation of the ETCC in studied areas.

Accordingly, carrying capacity has been defined by three indices, which relevantly resulted from several studies [44,58,59,60,61,62]: the PCC, RCC, and ECC, where each index is derived from a correction of the previous one. For example, the PCC is greater than the RCC, while the RCC may be greater or equal to the ECC Formula (1).

PCC > RCC and RCC ≥ ECC(1)

According to the methodology, the PCC has been defined as the maximum number of tourists that can visit from a specific destination during a given time Formula (2).

PCC = A × V/a × R_f_ (2)

where A is the area of the ecotourism zone (m^2^), V/a is the amount of space every ecotourism needs to be able to move freely (tourists/m^2^), and R_f_ is the number of permissible daily visits to a recreational destination (dividing the time of place availability by the average time of a visit) (unitless). The R_f_ has been determined to be 8 h/day, according to the period specified in each studied area.

The RCC has been defined as the maximum permissible number of visitors in an ecotourism destination when the limiting factors of the site have been applied to the PCC and formulated as follows Formulas (3) and (4):

RCC = PCC − C_f1_ − C_f2_ − … − C_fn_ (3)

C_f_ = (m/M_t_) × 100 (4)

Formula (5) explains the RCC with the corrective factors in percentages.

RCC= PCC × ((100 − C_f1_)/100) × ((100 − C_f2_)/100) × ((100 − C_fn_)/100) (5)

where PCC = physical carrying capacity, C_f_ = limiting factor, m = limiting value of a variable, and Mt = total value of variable.

Since the limiting variables depend on each area’s specific conditions and characteristics, according to Table 2, we identified and classified the climatic limiting variables using the nearest climatic stations related to each of the studied areas for a long period (from 1996–2020). In addition to the climatic limiting variables, other limiting factors were also studied, which had no impact on ecotourism in these areas.

The ECC has been defined as the maximum number of tourists in ecotourism destinations that existing management can support sustainably. Management capabilities include a set of conditions (policies, laws and regulations, tourism facilities and infrastructure, workforce for protection and management of these areas, etc.) that a site requires to achieve its goals and functions, formulated as Formulas (6) and (7). Since protective purposes are a higher priority in PAs, the number of environmental guardians per hectare has been considered the main important management factor in this study.

ECC = RCC × (100 − FM/100)(6)

FM = (Imc − Emc/Imc) × 100(7)

where FM is facility management, Imc is ideal management capacity, and Emc is existing management capacity.

## 4. Results

### 4.1. Impacts of Ecotourism in PAs

The Delphi method was repeated in three rounds, and the results were obtained in the form of three tables. Due to space limitations in the article, the results of round 3 have been demonstrated in the main article (Table 3), and the results of rounds 1 and 2 have been presented in the Supplementary Materials (Tables S1 and S2). Hence, according to Table S1, 40 impacts were extracted in the first round of the Delphi method, and in the second round, 38 impacts were responded to using the experts’ viewpoints (Table S2). In the first round of the Delphi method, the impacts of an increase in local commodities prices and a decrease in employment rates in other industries from economic-institutional dimensions were removed from the questions in the next rounds, according to the experts’ opinions. Furthermore, in the second round, the impact of an increase in the economic and employment damage from the economic-institutional dimensions was removed from the questions in the next rounds. Finally, in the third round, the impact of changes in the behavior of the local communities from the socio-cultural dimensions was removed from the questions, according to the experts’ viewpoints.

Accordingly, in round 3 of the Delphi method, 36 impacts were responded to by the experts, including 21 impacts in the environmental-physical dimensions, 8 impacts in the socio-cultural dimensions, and 7 impacts in the economic-institutional dimensions (Table 3). According to Table 3, the results indicated that most of the impacts of ecotourism were related to the environmental-physical dimensions, with a score of 3.38. In contrast, the lowest effects were related to the economic-institutional dimensions, with a score of 3.14. Hence, the results revealed that the most important impacts of ecotourism are different in the environmental-physical, demographic-social, and economic-institutional dimensions. In addition, a comparison of the means of the impacts of tourism activities in each dimension revealed that in the environmental-physical dimensions, the largest impact was related to the destruction of the habitat and ecosystem. In contrast, the smallest impact was related to the change in biogeochemical cycles. In the socio-cultural dimensions, the largest impact was associated with the increase of crime and insecurity, and the smallest impact was related to an increase in accidents. Finally, in the economic-institutional dimensions, the largest impact was related to the rise in inflation, with a score of 3.78. The most negligible impact was associated with the increase in demand for public services, such as health, security, police, and law enforcement, with a score of 2.53 (Table 3).

### 4.2. Extensive and Intensive Ecotourism

**Figure 4:**
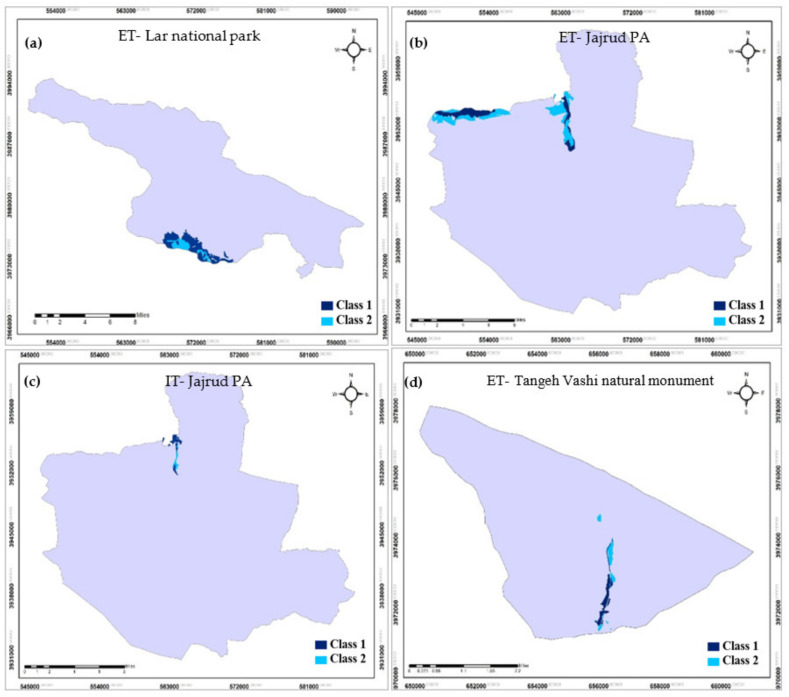
ET and IT maps in the studied areas; (**a**) Lar, (**b**) and (**c**) Jajrud, (**d**) Tangeh Vashi.

According to Table 4 and Figure 4, the results demonstrated that 1000.0 ha (2.8%) from this area in Lar national park was related to extensive ecotourism (ET). In the Jajrud PA, 728.0 ha was associated with ET and intensive ecotourism (IT). Finally, in the Tangeh Vashi natural monument, 5.3 ha was related to ET. Moreover, most ET was related to Lar national park, with 557.1 ha in class 2, while the least was related to Tangeh Vashi natural monument, with an area of 1.84 ha in class 1 (Table 4).

### 4.3. Analysis of ETCC

The PCC is estimated considering the two different amounts of space required by each ecotourist to move freely (2500 and 1500 m^2^ in ET and IT zones, respectively, according to the tourist’s opinions and the IUCN classification). According to Table 5, eight limiting variables have been calculated to estimate the RCC (according to Equation (3)). Accordingly, the most limiting climatic variables are related to Jajrud PA, with 84 d/y, while the least limiting climatic variables are related to Lar national park, with 58 d/y. According to the results, the highest RCC in the ET zone is related to Jajrud PA, with 2190 v/y in class 2, while the lowest RCC of the ET zone is related to Lar national park, with 109 v/y in class 1. Finally, the ECC has been estimated considering the management capacities related to staff, protection of infrastructure, and safety facilities in this study. Since the main factor in the management and protection of these areas is environmental guardians, this study has investigated their capacity. Therefore, according to the standards reported by the Department of Environment of Iran [53], one environmental guardian is required per 1000 ha of PA. According to the results, the highest ECC in the ET zone is related to Jajrud PA, with 1095 v/y in class 2, while the lowest ECC in the ET zone is related to Lar national park, with 109 v/y in class 1 (Table 5).

## 5. Discussion

As the studied areas require appropriate planning and management, ETCC has been used as a sustainable ecotourism planning and decision-making strategy in this study. Therefore, policies and methods of monitoring ETCC are essential in ecotourism destinations, especially in PAs. Hence, the obtained results can be reliable for decision-making and planning in PA management and achieving sustainability.

Although recreational use and carrying capacity are stated in the planning section of PA regulations, these regulations are not taken into account by the executive agencies during implementation. Accordingly, ETCC has been used as a valuable tool in PA management and planning for optimal ecotourism destinations and the prevention and control of the negative impacts of ecotourism in these areas. The results revealed that in Lar national park, the highest number of tourists was related to the PCC, while the lowest number of tourists was observed in the ECC. Accordingly, many factors have been caused to decrease the PCC and increase the unsustainable ecotourism in this area, such as insufficient monitoring by the Provincial Department of Environment, monitoring mechanisms, the low number of guard stations, the number of environmental guardians, the amount of protective equipment against fire, illegal harvesting, and overgrazing (e.g., fire protective garments and unmanned aerial vehicles), insufficient training, and livestock overgrazing. One of the most critical threats in this area is an excessive number of tourists and uncontrolled livestock entry in this area, which has led to the destruction of vegetation and threatened wildlife habitats in this area. Jahani and Saffariha [72] assessed the impacts of livestock and ecotourism on vegetation in Lar national park. Their results indicated that vegetation diversity has decreased due to overgrazing and the development of ecotourism activities. Livestock grazing can reduce plant richness and vegetation cover [73]. Törn et al. [74] also confirmed the impacts of ecotourism activities on vegetation in the natural areas of Finland, and the results indicated that these impacts lead to delays in the restoration of vascular plant species. Moreover, many studies suggest that the diversity of flora and fauna species has decreased due to the development of human activities in PAs [75,76,77,78,79]. Their results will contribute to a greater understanding of the impacts of ecotourism management on the sustainability of the national parks and PAs. Hence, in Lar national park, it is necessary to manage and control the number of tourists, according to the potential and the ETCC of the area. Moreover, the poaching pressures of the tourists in Lar national park must be reduced to prevent extirpations of large- and medium-sized mammals [80]. Pervious t research highlighted that poaching by tourists increases wildlife mortality rates and can reduce the abundance and number of mammalian species in PAs [81,82], leading to habitat degradation.

As the results revealed in Jajrud PA, the highest number of tourists was related to the PCC, while the lowest number of tourists was observed in the ECC. Moreover, the highest IT was related to the PCC, while the lowest was related to the RCC and ECC modes. Accordingly, the number of tourists in the ET zone was more than the number of tourists in the IT zone due to the priorities of conserving and protecting biodiversity in this area and considering management capacities related to the staff and protection infrastructures. In Jajrud PA, due to insufficient monitoring, ecotourism activities have brought destruction to the natural resources of this area, and undoubtedly, the continuation of this trend will increase the unsustainability level [4]. Moreover, one of the main reasons for the decreased vegetation and loss of biodiversity in this area is the excessive presence of tourists and the uncontrolled development of ecotourism activities. Another important factor leading to the increasing development of ecotourism activities in this area is the numerous national and regional attractions, which encourage tourists to visit this area and promote the development of unplanned ecotourism activities. Furthermore, a low level of organizational cooperation, a low level of awareness among tourists and the local communities, weakness in some rules and regulations, and a lack of implementation of ecotourism management designs have led to extensive destruction and unsustainability in this area. These findings have been confirmed by Belsoy et al. [83] in Kenya. Their results indicated that ecotourism activities, directly and indirectly, have brought destruction to the natural resources in PAs and can determine the degree of sustainability of sustainable ecotourism and the related activities in these areas. In addition, these findings have been confirmed in other studies in Iran [84], Italy [15], and Mexico [65], which have demonstrated habitat destruction and the development of ecotourism unsustainability. As this area requires appropriate planning and management, the continuation of these activities without the proper planning and managerial strategies will lead to the further destruction of ecosystems, the extinction of species, and unsustainability in Jajrud PA.

In Tangeh Vashi, the highest number of tourists was related to the PCC, while the lowest number of tourists was observed in the RCC and ECC modes. One of the most critical issues that threatened the habitat was related to a large number of tourists in this area, especially on weekends and holidays. According to statistics from the Department of the Environment of Iran [53], more than 300,000 tourists visit Tangeh Vashi annually, and this issue has led to a decrease in vegetation density, animal migration, and habitat destruction. Other critical factors that have led to increased destruction and unsustainability in this area include a lack of participation between local people and tourist leaders and low levels of awareness and education of tourists and local communities. In addition, the most important issues affecting the development of uncontrolled ecotourism were the lack of monitoring and control of the area, inadequate number of guard stations, environmental guardians to monitor the density of tourists, and insufficient infrastructure and ecotourism facilities. Other studies have confirmed these findings in Nepal [10] and China [85], demonstrating this natural ecosystem’s destructive intensity and unsustainability trend.

Among the ETCCs in these areas, the highest PCC was related to Lar national park, and the lowest was associated with Tangeh Vashi natural monument. As stated in the Materials and Methods, the PCC depends on the available space for visitors and the duration (opening time) of the visit [44]. The Lar national park had the highest PCC because of its relatively easy access for tourists, and this finding is similar to previous research in Portugal [86]. Moreover, the highest RCCs and ECCs were related to Jajrud PA. The RCC index is capable of identifying the visitors’ impact in affecting the physical factors for PA management [87]. According to tourism management, the RCC value with the lowest value limits tourism operations [43,87]. The ECC index is based on the number and capacity of the existing staff in providing services to tourists, and it is the maximum number of visitors that will not disrupt the attraction and can be handled by the current staff members with the available management system [87].

## 6. Conclusions

Undoubtedly, ecological sustainability has a relevant relationship with the capacity of ecotourism destinations and tourists’ satisfaction, so inattention to this subject can lead to unsustainability in PAs. The results revealed that, despite the ecological sensitivity of these areas, excessive ecotourism had increased the destruction and unsustainability in the ecosystems of these areas. The current findings demonstrated that the intensity of destruction varies between the studied areas, due to distance from the metropolis of Tehran and the development of ecotourism, including an increasing requirement of urban communities to develop ecotourism, insufficient facilities and infrastructures, population growth, increasing urbanization, and other functions.

To sum up, national parks and national natural monuments have a high sensitivity and low flexibility to develop human activities. Accordingly, in Jajrud PA, the number of tourists is more than the RCC and ECC, due to the low level of protection restrictions and legal instructions. In contrast, in Lar national park and Tangeh Vashi natural monument, due to the short duration of tourism-related weather conditions (from June to October), high level of restrictions, and ecological sensitivity, the number of tourists is less than the RCC and ECC. The lowest ECC is related to the Lar national park and Tangeh Vashi natural monument, due to the number of environmental guardians and the monitoring level. Therefore, protecting these areas requires the implementation of management strategies to control the negative impacts of ecotourism and estimate the number of visitors.

### 6.1. Theoretical Contribution

Due to a change in travel ideas, ecotourism has grown rapidly. A common approach to minimizing environmental degradation is estimating the ETCC and setting up visitor access limitations. This research extends the current knowledge on the ETCC with some critical research dimensions in various PAs located in the most populous province of Iran. This study investigated the ecotourism potential and estimated the ETCC in PAs of Tehran province to minimize the negative impacts of ecotourism in different dimensions, including environmental-physical, demographic-social, and economic-institutional.

### 6.2. Practical Implications

1. For the government: It is necessary to control the rising trend of unplanned ecotourism and unsustainability in PAs. It is required to pay attention to various factors, including organizational cooperation, awareness among tourists and local communities, the quality of governmental and non-governmental institutional performance in training and information, an increase of non-governmental organizations (NGOs), local councils, institutions, service enterprises, planning and implementation of ecotourism management designs, etc. Moreover, the development of intensive recreation zones must be minimized and established outside the border of the areas (i.e., far from the biologically sensitive areas). Therefore, developing ecotourism in these areas requires presenting integrated ecotourism management plans, increasing monitoring mechanisms, and increasing the cooperation and partnership between government organizations and other government agencies.
2. For tourism enterprises: They should take social responsibility, have a long-term vision, and respond to the government’s call to implement rural ecotourism projects for residents to better serve local revitalization.
3. For residents: Local residents, with the help of the government and tourism enterprises, should change their traditional concepts, become familiar with the relevant rural ecotourism policies, and actively participate in rural ecotourism projects.

### 6.3. Research Limitations and Future Research

In addition to its several theoretical and practical implications, this study also has several limitations that could be examined in future studies. Some of the future research directions have been presented, which include evaluating the negative impacts of ecotourism management policies in PAs, assessing “Environmental Impact Assessment (EPA)” to avoid the adverse effects of ecotourism development in these areas, ecotourism impact prediction using machine learning models (e.g., artificial neural networks (ANNs) and support vector machines (SVM)), and meta-analysis of the controlling strategies of ecological destruction and the negative impacts of ecotourism.

## Figures and Tables

**Table 1:** Description of the three study areas in Tehran province.

Site	Area (ha)	History of Protected Area	Distance from Tehran City	Total Number of Environmental Guardians	Number of Environmental Guardiansper Hectare (%)	Number of Guard Stations	Number of Tourists (Visitor/ Year)
Lar national park	35,765	Established in 2001	70 Km	14	0.04	4	36,000
Jajrud protected area with the sustainable use of natural resources	74,811	Established in 1982	Located in Tehran	28	0.04	7	50,000
Tangeh Vashi natural monument	3650	Established in 2011	160 km	4	0.11	1	300,000

**Table 2:** List of climatic limiting variables in the studied areas.

Site	Meteorological Station Information				Limiting Variable							
	Name	Elevation (m a.s.l.)	Latitude (*N*)	Longitude (*E*)	C_f1_	C_f2_	C_f3_	C_f4_	C_f5_	C_f6_	C_f7_	C_f8_
Lar national park	Damavand Synoptic	2051	35° 43′ 00″	52° 03′ 00″	12	3	4	12	57	2	2717	42
Jajrud PA	Lavasan Synoptic	1863	35° 49′ 54″	51° 38′ 33″	19	15	10	25	116	3	2833	49
Tangeh Vashi natural monument	Firuzkooh Synoptic	1976	35° 45′ 00″	52° 44′ 00″	15	2	4	11	144	3	3010	24

where the climatic limiting variables: C_f1_ = the number of days with lightning, C_f2_ = the number of days with fog, C_f3_ = the number of days with dust, C_f4_ = the number of days with visibility less than 2000 m, C_f5_ = the number of days with frost, C_f6_ = the number of days with an average cloud level, C_f7_ = the number of days with intense sunny hours, and C_f8_ = the number of days with maximum wind speed.

**Table 3:** Results of round 3 of the Delphi method about the negative impacts of ecotourism in the studied areas.

Dimensions	Impacts	*n*	Mean	SD	V	Rank	Total Mean
Environmental-physical	(1) Destruction of the habitat and ecosystem	33	4.32	1.000	2.000	1	3.38
	(2) Extinction of biologically valuable species (fauna and flora)	33	4.26	1.000	1.060	2	
	(3) Increase in wildlife hunting	33	3.21	1.000	1.000	13	
	(4) Change of the wildlife species’ diet and their migration path	33	3.16	1.000	2.030	14	
	(5) Reduction and loss of vegetation covers	33	4.11	1.000	1.000	3	
	(6) Change in the ecosystem function (flow of matter, energy and information, etc.)	33	3.08	1.000	1.000	15	
	(7) Decrease in biodiversity	33	3.86	1.000	1.000	4	
	(8) Decrease in ecosystem services	33	3.72	1.030	2.000	6	
	(9) Decrease in natural resources	33	3.76	1.000	2.055	5	
	(10) Increase in environmental pollution	33	3.68	1.000	1.000	7	
	(11) Decrease in the reservoirs of groundwater aquifers and a change in the surface water regime	33	3.57	1.000	1.000	8	
	(12) Increase in climate change	33	3.48	1.000	1.000	9	
	(13) Change in biogeochemical cycles	33	2.63	1.000	1.000	21	
	(14) Increase in the water evaporation level	33	2.68	1.000	1.000	20	
	(15) Increase in the soil erosion level	33	3.31	1.000	1.000	11	
	(16) Increase in the LULC changes for the development of tourism infrastructure	33	3.35	1.029	2.000	10	
	(17) Disturbance of landscape	33	3.27	1.000	1.060	12	
	(18) Increase in garbage per visitor	33	2.94	1.000	1.000	17	
	(19) Changes in the quality of local services	33	3.02	1.029	1.043	16	
	(20) Increase in abrupt environmental crises (such as storms, floods, and earthquakes)	33	2.77	1.000	1.000	19	
	(21) Increase in the congestion in roads and public places	33	2.85	1.029	1.000	18	
Socio-cultural	(1) Increase in crime and insecurity	33	3.84	1.000	2.030	1	3.32
	(2) Increase in accidents	33	2.71	1.000	1.000	8	
	(3) Destruction of cultural-historical and ancient monuments	33	3.46	1.000	1.000	4	
	(4) Changes in the culture of local communities	33	3.31	1.000	1.000	5	
	(5) Dissatisfaction in local communities	33	3.57	1.000	2.000	3	
	(6) Increase in cultural invasions	33	3.68	1.000	2.055	2	
	(7) Changes in quality of life standards	33	3.16	1.029	1.000	6	
	(8) Increase in diseases	33	2.88	1.000	1.000	7	
Economic-institutional	(1) Increase in taxes on land, buildings, and other structures	33	3.62	1.000	1.000	2	3.14
	(2) Increase in inflation	33	3.78	1.029	1.000	1	
	(3) Increase in the demand for public services (such as health, security, and police)	33	2.53	1.000	1.000	7	
	(4) Increase in the demand for economic infrastructure	33	2.67	1.000	1.000	6	
	(5) Increase in tourism costs	33	3.11	1.000	1.000	4	
	(6) Increase in seasonal employees in tourism	33	2.95	1.000	1.000	5	
	(7) Increase in economic pressures on households	33	3.34	1.000	1.000	3	

**Table 4:** Extensive ecotourism (ET) and intensive ecotourism (IT) recreation classes for the studied areas.

Site	ET/IT	Classes	Area		Total Area	
			ha	%	ha	%
Lar national park (total area = 35,765 ha)	ET	1	442.9	44.3	1000.0	2.8
		2	557.1	55.7		
Jajrud PA (total area = 74,811 ha)	ET	1	286.2	39.3	728.0	0.1
		2	441.8	60.7		
	IT	1	11.0	55.8	19.8	0.03
		2	8.8	44.2		
Tangeh Vashi natural monument (total area = 3,650 ha)	ET	1	1.8	35.0	5.3	0.1
		2	3.4	65.0		

**Table 5:** Estimation of the physical carrying capacity (PCC), real carrying capacity (RCC), and effective carrying capacity (ECC) for the studied areas.

Carrying Capacity	Site	Recreation Class	Parameter											Number of Visitors	
			A (m^2^)					R_f_ (h)		V/a (m^2^)				(Day)	(Year)
PCC	Lar national park	ET1	4,430,300					8		2500				1772	193,148
		ET2	5,566,100											22,226	242,634
	Jajrud PA	ET1	2,858,000					8		2500				1143	417,195
		ET2	4,422,000											1768	645,320
		IT1	112,600					8		1500				75	27,375
		IT2	88,500											59	21,535
	Tangeh Vashi natural monument	ET1	18,500					8		2500				7	1085
		ET2	34,700											13	2015
		**Recreation Class**	**Limiting Variables**											**Number of Visitors**	
			**C_f1_**	**C_f2_**	**C_f3_**		**C_f4_**		**C_f5_**	**C_f6_**	**C_f7_**	**C_f8_**	**C_fn_**	**(day)**	**(year)**
RCC	Lar national park	ET1	3	1	1		3		16	1	21	12	58	1	109
		ET2												2	218
	Jajrud PA	ET1	5	4	3		7		32	1	19	13	84	4	1460
		ET2												6	2190
		IT1												1	365
		IT1												2	730
	Tangeh Vashi natural monument	ET1	4	1	1		3		39	1	18	7	74	1	155
		ET2												3	465
		**Recreation Class**	**Management Factors**											**Number of Visitors**	
			**Imc**			**Emc**					**FM**			**(day)**	**(year)**
ECC	Lar national park	ET2	36			14					61			1	109
	Jajrud PA	ET1	75			28					63			2	730
		ET2												3	1095
		IT2												1	365
	Tangeh Vashi natural monument	ET1	5			4					20			1	155
		ET2												2	310

Note: A—Area of the ecotourism zone; R_f_—The ratio of the usable time of the area to the average length of the visit time; V—The value equal to 1 visitor; a shows the amount of space required by each visitor; Imc—Ideal management capacity; Emc—Existing management capacity; FM—Facility management.

## Data Availability

The data that support the findings of this study are available from the corresponding author (H.E.), upon reasonable request.

## Supplementary Materials

The following are available online at https://www.mdpi.com/article/10.3390/ijerph19031059/s1, Table S1: Results of round 1 of the Delphi method about the negative impacts of tourism activities in PAs, Table S2: Results of round 2 of the Delphi method about the negative impacts of tourism activities in PAs.
