# Supplementary material for: Estimation of Ecotourism Carrying Capacity for Sustainable Development of Protected Areas in Iran
Source: Int J Environ Res Public Health. 2022 Jan 18;19(3):1059. doi: 10.3390/ijerph19031059 (PMC8834276; doi:10.3390/ijerph19031059)
Supplement: Supplementary file 1 [file ijerph-19-01059-s001.zip › ijerph-1512019-supplementary.pdf]

Supplementary Materials

Table S1. Results of round 1 of the Delphi method about the negative impacts of tourism activities in PAs.

Dimensions	Impacts	*n*	Mean	SD	V	Rank	Total Mean
Environmental-physical	(1) Destruction of the habitat and ecosystem	38	4.21	1.000	2.000	1	3.28
	(2) Extinction of biologically valuable species (fauna and flora)	38	4.15	1.000	2.055	2	
	(3) Increase in wildlife hunting	38	3.14	1.000	1.000	13	
	(4) Change of the species' diet and their migration path	38	3.04	1.000	1.000	14	
	(5) Reduction and loss of vegetation covers	38	4.02	1.000	1.000	3	
	(6) Change in the ecosystem function (flow of matter, energy, and information, etc.)	38	3	1.029	1.000	15	
	(7) Decrease in biodiversity	38	3.74	1.000	1.000	4	
	(8) Decrease in ecosystem services	38	3.62	1.000	1.000	6	
	(9) Decrease in natural resources	38	3.68	1.000	2.000	5	
	(10) Increase in environmental pollution	38	3.62	1.000	1.060	7	
	(11) Decrease in the reservoirs of groundwater aquifers and a change in the surface water regime	38	3.48	1.000	1.000	8	
	(12) Increase in climate change	38	3.4	1.000	2.000	9	
	(13) Change in biogeochemical cycles	38	2.51	1.030	2.055	21	
	(14) Increase in the water evaporation level	38	2.58	1.000	1.000	20	
	(15) Increase in the soil erosion level	38	3.24	1.000	1.000	11	
	(16) Increase in the LULC changes for the development of tourism infrastructure	38	3.28	1.000	1.000	10	
	(17) Disturbance of landscape	38	3.19	1.000	1.000	12	
	(18) Increase in garbage per visitor	38	2.86	1.000	1.000	17	
	(19) Changes in the quality of local services	38	2.88	1.000	1.000	16	
	(20) Increase in abrupt environmental crises (such as storms, floods, and earthquakes)	38	2.66	1.000	2.000	19	
	(21) Increase in the congestion in roads and public places	38	2.77	1.029	1.060	18	
Socio-cultural	(1) Increase in crime and insecurity	38	3.74	1.000	2.000	1	3.18
	(2) Increase in accidents	38	2.66	1.000	1.060	8	
	(3) Destruction of the cultural-historical and ancient monuments	38	3.4	1.000	1.000	4	
	(4) Changes in the culture of local communities	38	3.28	1.000	2.030	5	
	(5) Dissatisfaction in local communities	38	3.51	1.030	1.000	3	
	(6) Increase in cultural invasions	38	3.55	1.000	1.000	2	
	(7) Changes in behavior of local communities	38	2.6	1.000	1.000	9	
	(8) Changes in quality of life standards	38	3.05	1.000	2.000	6	
	(9) Increase in diseases	38	2.82	1.000	2.055	7	
Economic - institutional	(1) Increase in taxes on land, buildings, and other structures	38	3.55	1.000	1.000	2	2.86
	(2) Increase in local commodities price	38	2.34	1.029	1.000	10	
	(3) Increase in inflation	38	3.72	1.000	1.000	1	
	(4) Increase in the demand for public services (such as health, security, and police)	38	2.45	1.000	1.000	7	
	(5) Increase of demand for economic infrastructure	38	2.61	1.000	1.000	6	
	(6) Increase in tourism costs	38	3.02	1.000	2.000	4	
	(7) Increase in seasonal employees in tourism	38	2.88	1.000	1.060	5	
	(8) Decrease in employment rates in other industries	38	2.4	1.000	1.000	8	
	(9) Increase in economic and employment damage	38	2.37	1.000	2.030	9	
	(10) Increase in economic pressures on households	38	3.27	1.000	1.000	3	

Table S2. Results of round 2 of the Delphi method about the negative impacts of tourism activities in PAs.

Dimensions	Impacts	*n*	Mean	SD	V	Rank	Total Mean
Environmental-physical	(1) Destruction of the habitat and ecosystem	35	4.28	1.000	2.000	1	3.32
	(2) Extinction of biologically valuable species (fauna and flora)	35	4.18	1.000	2.055	2	
	(3) Increase in wildlife hunting	35	3.17	1.000	1.000	13	
	(4) Change of the species' diet and their migration path	35	3.09	1.030	1.000	14	
	(5) Reduction and loss of vegetation covers	35	4.08	1.000	1.000	3	
	(6) Change in the ecosystem function (flow of matter, energy, and information, etc.)	35	3.02	1.000	1.000	15	
	(7) Decrease in biodiversity	35	3.78	1.000	1.000	4	
	(8) Decrease in ecosystem services	35	3.68	1.000	1.000	6	
	(9) Decrease in natural resources	35	3.71	1.000	2.000	5	
	(10) Increase in environmental pollution	35	3.64	1.000	1.060	7	
	(11) Decrease in the reservoirs of groundwater aquifers and a change in the surface water regime	35	3.52	1.000	1.000	8	
	(12) Increase in climate change	35	3.44	1.000	2.000	9	
	(13) Change in biogeochemical cycles	35	2.55	1.000	2.055	21	
	(14) Increase in the water evaporation level	35	2.61	1.000	1.000	20	
	(15) Increase in the soil erosion level	35	3.27	1.000	1.000	11	
	(16) Increase in the LULC changes for the development of tourism infrastructure	35	3.32	1.029	1.000	10	
	(17) Disturbance of landscape	35	3.22	1.000	1.000	12	
	(18) Increase in garbage per visitor	35	2.88	1.000	1.000	17	
	(19) Changes in the quality of local services	35	2.91	1.000	1.000	16	
	(20) Increase in abrupt environmental crises (such as storms, floods, and earthquakes)	35	2.68	1.000	2.000	19	
	(21) Increase in the congestion in roads and public places	35	2.8	1.000	1.000	18	
Socio-cultural	(1) Increase in crime and insecurity	35	3.8	1.000	1.000	1	3.22
	(2) Increase in accidents	35	2.68	1.030	1.000	8	
	(3) Destruction of cultural-historical and ancient monuments	35	3.44	1.000	1.000	4	
	(4) Changes in the culture of local communities	35	3.3	1.000	1.000	5	
	(5) Dissatisfaction in local communities	35	3.54	1.000	2.000	3	
	(6) Increase in cultural invasions	35	3.61	1.000	1.000	2	
	(7) Changes in behavior of local communities	35	2.66	1.000	1.000	9	
	(8) Changes in quality of life standards	35	3.11	1.000	1.000	6	
	(9) Increase in diseases	35	2.84	1.000	2.000	7	
Economic - institutional	(1) Increase in taxes on land, buildings, and other structures	35	3.58	1.029	2.055	2	3.02
	(2) Increase in inflation	35	3.75	1.000	1.000	1	
	(3) Increase in the demand for public services (such as health, security, and police)	35	2.48	1.000	1.000	7	
	(4) Increase in the demand for economic infrastructure	35	2.64	1.000	1.000	6	
	(5) Increase in tourism costs	35	3.09	1.000	1.000	4	
	(6) Increase in seasonal employees in tourism	35	2.91	1.000	1.000	5	
	(7) Increase in economic and employment damage	35	2.44	1.029	1.000	8	
	(8) Increase in economic pressures on households	35	3.3	1.000	2.000	3	
